# A genomics resource for genetics, physiology, and breeding of West African sorghum

**DOI:** 10.1002/tpg2.20075

**Published:** 2021-04-05

**Authors:** Jacques M. Faye, Fanna Maina, Eyanawa A. Akata, Bassirou Sine, Cyril Diatta, Aissata Mamadou, Sandeep Marla, Sophie Bouchet, Niaba Teme, Jean‐Francois Rami, Daniel Fonceka, Ndiaga Cisse, Geoffrey P. Morris

**Affiliations:** ^1^ Department of Agronomy Kansas State University Manhattan KS USA; ^2^ Institut Sénégalais de Recherches Agricoles Centre d’Étude Régional pour l'Amélioration de l'Adaptation à la Sécheresse Thies Senegal; ^3^ Institut National de la Recherche Agronomique du Niger Niamey Niger; ^4^ Institut Togolaise de Recherche Agronomique Lomé Togo; ^5^ Institut d'Economie Rurale BP 258, Rue Mohamed V, Bamako Mali; ^6^ Genetic Improvement and Adaptation of Mediterranean and Tropical Plants, Montpellier University CIRAD, INRA, Montpellier SupAgro Montpellier France; ^7^ The French Agricultural Research Centre for International Development, CIRAD, UMR AGAP BP Thies 3320 Senegal

## Abstract

Local landrace and breeding germplasm is a useful source of genetic diversity for regional and global crop improvement initiatives. Sorghum (*Sorghum bicolor* L. Moench) in western Africa (WA) has diversified across a mosaic of cultures and end uses and along steep precipitation and photoperiod gradients. To facilitate germplasm utilization, a West African sorghum association panel (WASAP) of 756 accessions from national breeding programs of Niger, Mali, Senegal, and Togo was assembled and characterized. Genotyping‐by‐sequencing (GBS) was used to generate 159,101 high‐quality biallelic single nucleotide polymorphisms (SNPs), with 43% in intergenic regions and 13% in genic regions. High genetic diversity was observed within the WASAP (π = .00045), only slightly less than in a global diversity panel (GDP) (π = .00055). Linkage disequilibrium (LD) decayed to background level (*r^2^* < .1) by ∼50 kb in the WASAP. Genome‐wide diversity was structured both by botanical type and by populations within botanical type with eight ancestral populations identified. Most populations were distributed across multiple countries, suggesting several potential common gene pools across the national programs. Genome‐wide association studies (GWAS) of days to flowering (DFLo) and plant height (PH) revealed eight and three significant quantitative trait loci (QTL), respectively, with major height QTL at canonical height loci *Dw3* and *SbHT7.1*. Colocalization of two of eight major flowering time QTL with flowering genes previously described in U.S. germplasm (*Ma6* and *SbCN8*) suggests that photoperiodic flowering in West African sorghum is conditioned by both known and novel genes. This genomic resource provides a foundation for genomics‐enabled breeding of climate‐resilient varieties in WA.

## Core Ideas

- A West African sorghum panel (*n* = 756) was assembled from four national programs.
- Over 150,000 genome‐wide nucleotide polymorphisms were genotyped by sequencing.
- Diversity was structured by subpopulation within botanical type and across countries.
- Known genes and novel loci for flowering time and plant height were identified.

**Abbreviations**
BLUP: best linear unbiased prediction
DFLo: days to flowering
F_ST_
: fixation index
GBS: genotyping‐by‐sequencing
GDP: global sorghum diversity panel
GLM: general‐linear model
GWAS: genome‐wide association study
LD: linkage disequilibrium
MAF: minor allele frequency
MLMM: multi‐locus mixed model
PC: principal component
PCA: principal component analysis
PH: plant height
PVE: proportion of variance explained
QTL: quantitative trait loci
SNP: single nucleotide polymorphism
WA: western Africa
WAS‐GDP: West African accessions in the global sorghum diversity panel
WAS‐GRIN: West African accessions from USDA–GRIN
WASAP: West African sorghum association panel.

## 1 INTRODUCTION

Crop production in many developing countries is limited by biotic and abiotic factors that reduce food supplies to small‐holder farmers in semiarid areas. With an increasing worldwide underfed population along with environmental changes, there is a need in more rapidly developing locally adapted varieties to increase crop productivity (Foley et al., 2011; Mundia, Secchi, Akamani, & Wang, 2019; Tilman, Balzer, Hill, & Befort, 2011). Genetic studies contribute to the development of adapted cultivars to meet global food security and help provide enough genetic diversity suitable for efficient crop breeding (Jordan, Mace, Cruickshank, Hunt, & Henzell, 2011). Diverse landrace germplasm harbors useful alleles for gene discovery and breeding given their long history of adaptation to diverse environments (Meyer & Purugganan, 2013). However, African crop genetic diversity, particularly in western Africa (WA), are poorly characterized mainly as a result of a lack of genomic resources and limited sampling of genetic resources available to the global scientific community.

Understanding genomic variation of local germplasm at a regional scale can help guide breeding. The availability of high‐density markers evenly distributed throughout the genome is a prerequisite for understanding genetic diversity and genetic basis of adaptive traits. Recent advances in next‐generation sequencing technologies and genotyping‐by‐sequencing (GBS) have rendered possible the generation of high‐density markers with affordable low cost (Elshire et al., 2011; Poland, Brown, Sorrells, & Jannink, 2012). These tools facilitate characterization of the genetic structure of local germplasm relative to global diversity. Historical recombination along with short to moderate linkage disequilibrium (LD) existing within a diversity panel greatly improve the mapping resolution to identify novel genes and novel natural variants at known genes in major crops (Cao et al., 2016; Gapare et al., 2017; Huang et al., 2010; Yano et al., 2016; Zhao et al., 2019).

In sorghum, global reference diversity panels have been assembled (Grenier, Hamon, & Bramel‐Cox, 2001; Deu, Rattunde, & Chantereau, 2006; Casa et al., 2008; Upadhyaya et al., 2009; Billot et al., 2013; Brenton et al., 2016) and used in genetic and breeding studies. However, at a regional scale, these global reference panels have limited population sampling, thus limiting their potential use in regional association mapping studies. Regional diversity panels are useful genetic resources for capturing natural allelic variation existing in locally adapted varieties (Leiser et al., 2014; Sattler et al., 2018). Favorable alleles for adaptation to various regional environmental conditions have been selected for over thousands of years but they may not be easily accessible to breeders via global ex situ collections (Fu, 2017; Hammer & Teklu, 2008). The major western and central African cereal crops, such as sorghum and pearl millet [*Cenchrus americanus* (L.) Morrone], need to be assembled into regional reference diversity panels that can be accessed for genetics, physiology, and breeding.

Genetic diversity of cultivated sorghum is high in West African germplasm (Deu et al., 1994; Doggett, 1988; Folkertsma, Rattunde, Chandra, Raju, & Hash, 2005). Despite the high genetic diversity in the West African germplasm, its accessibility to the regional and global scientific community is limited, particularly germplasm that was more recently collected or developed. Five botanical types in sorghum—bicolor, durra, guinea, caudatum and kafir—and 10 intermediate types have been defined based on spikelet and grain morphology (Harlan & De Wet, 1972) and are associated with climate and geographic origin (Brown, Myles, & Kresovich, 2011). All botanical types are represented in WA landraces except for the kafir type. The guinea type is the most common and diverse in WA, possibly a result of a second center of domestication (Folkertsma et al., 2005). The durra type was first domesticated in Ethiopia before diffusing to arid regions of WA. The caudatum and durra–caudatum intermediate types are well represented in the western–central region and used for grain yield improvement in breeding programs throughout WA (Institut sénégalais de recherches agricoles, 2005). However, little is known about the population structure of the germplasm across the countries, that is, whether the germplasm of one country is distinct from other countries. Since country boundaries in WA cut across agro‐ecological zones, some landrace germplasm may be more similar across countries than within a country.

**Core Ideas**

- A West African sorghum panel (*n* = 756) was assembled from four national programs.
- Over 150,000 genome‐wide nucleotide polymorphisms were genotyped by sequencing.
- Diversity was structured by subpopulation within botanical type and across countries.
- Known genes and novel loci for flowering time and plant height were identified.

Here, we report the assembly of the West African sorghum association panel (WASAP) from the four West African countries (Mali, Niger, Senegal, and Togo) and development of genome‐wide SNP markers as genomic resources for genetics, physiology, and breeding. We determined the genome‐wide SNP variation and population structure of the germplasm in relationship with previously genotyped West African *ex situ* collections and global sorghum diversity panel (GDP). We also identified known maturity and plant height (PH) loci using GWAS on phenotype data collected under rainfed conditions. The study provides genomic resources and a better understanding of the population structure of the WA germplasm useful for genomics‐assisted breeding.

## 2 MATERIALS AND METHODS

### 2.1 Plant materials

The WASAP was comprised of 845 accessions assembled by breeders, physiologists, and geneticists in the National Agricultural Research Organization from four West African countries (Senegal, Mali, Togo, and Niger) (Table 1; Supplemental Data S1). The panel includes working collections of the National Agricultural Research Organization sorghum improvement programs, predominantly landraces but also improved varieties and breeding lines (*n* = 72). Seed multiplication was performed for these accessions under rainfed conditions of 2014. Out of the 845 accessions, 756 accessions were genotyped using GBS, with 118 accessions from Senegal, 123 accessions from Mali, 156 accessions from Togo, and 359 accessions from Niger. The genotyped accessions were used for genetic analyses in this study. Based on a priori classification, all four basic botanical types—guinea (*n* = 244), caudatum (*n* = 72), durra (*n* = 23), and bicolor (*n* = 22)—are well represented in the WASAP. The guinea margaritiferum is represented by nine accessions. The intermediate types are well represented: durra–caudatum (*n* = 161), followed by guinea–caudatum (*n* = 25), durra–bicolor (*n* = 13), caudatum–bicolor (*n* = 8), guinea–durra (*n* = 5), and kafir–caudatum (*n* = 1). Many accessions were not classified morphologically into botanical classes (*n* = 230). The Supplemental Data S1 contains more details about the panel.

**TABLE 1:** Number of accessions in each sorghum collection/panel used in this study

	Collection or panel^a^		
Country of origin	WASAP	WAS‐GRIN	GDP
Mali	123	–	15
Niger	359	515	12
Senegal	118	346	8
Togo	156	–	3
Gambia	–	60	4
Mauritania	–	15	–
Nigeria	–	607	38
Other western Africa	–	–	18
Total from western Africa	756	1543	98
Central Africa	–	–	44
Northern Africa	–	–	10
Southern Africa	–	–	146
Eastern Africa	–	–	223
Middle East	–	–	37
Eastern Asia	–	–	26
Southern Asia	–	–	99
Other	–	–	9
Total	756	1543	692

^a^ WASAP, West African sorghum association panel; WAS‐GRIN, West African sorghum in USDA–GRIN; GDP, global sorghum diversity panel.

Genetic diversity and structure of the WASAP were compared with the GDP that consists of 692 worldwide sorghum accessions (excluding accessions from Americas) with available sequencing data including West African accessions in the GDP (hereafter named WAS‐GDP) (Lasky et al., 2015; Morris et al., 2013). The WASAP accessions were also compared with previously genotyped West African accessions from USDA–GRIN (hereafter named WAS‐GRIN), originating from Niger, Senegal (including a few accessions from neighboring Gambia and Mauritania), and Nigeria (Faye et al., 2019; Maina et al., 2018; Olatoye, Hu, Maina, & Morris, 2018).

### 2.2 Genotyping‐by‐sequencing and SNP discovery

The WASAP was grown in the field at the Bambey research station in Senegal, and leaf tissue from five seedlings of each accession were pooled to extract genomic DNA using the MATAB (mixed alkyl trimethylammonium bromide) protocol. Genotyping‐by‐sequencing was conducted following the method previously described (Elshire et al., 2011). Briefly, GBS libraries were constructed in 96‐plex and the restriction enzyme*, Ape*KI (New England Biolabs) was used to digest genomic DNA for complexity reduction. For quality control, a random well was left blank in each 96‐well plate. Restriction cutting sites were ligated using bar‐coded adapters and ligated products were pooled together. Single‐end sequencing was performed on Illumina HiSeq2500 at the University of Kansas Medical Center.

Illumina single‐end sequence reads of the WASAP and raw sequence data of the GDP were processed together using the TASSEL GBS v2 pipeline (Glaubitz et al., 2014). Sequence reads were trimmed to 64 bp and identical reads collapsed into tags. Tags were aligned to the sorghum reference genome v3.1 (McCormick et al., 2018; Paterson et al., 2009) using the Burrows–Wheeler alignment program (Li & Durbin, 2009). Single nucleotide polymorphism markers were discovered using the *DiscoverySNPCallerPluginV2* of the TASSEL GBS v2 pipeline with minimum locus coverage (*mnLov*) of 0.1 and other parameters kept to default settings. A total of 546,133 SNPs were discovered. The SNPs with >20% missing data were excluded (*n* = 393,396 remaining SNPs). The SNPs with minor allele frequency (MAF) < .01 were excluded (*n* = 201,193 remaining SNPs). Monomorphic sites were excluded and biallelic SNPs only were maintained, resulting in a total set of 198,402 SNPs. This dataset was imputed using Beagle v4.1 (Browning & Browning, 2016) and filtered out again data with MAF < .01 to yield a final data set of 159,101 SNPs that was used for downstream analysis.

### 2.3 Genome‐wide SNP variation

The imputed dataset was functionally annotated to determine SNP effects on protein‐coding genes using the snpEff program (Cingolani et al., 2012). The structural location and functional class (synonymous, missense, or nonsense) of each SNP were determined based on the sorghum reference sequence. Genome‐wide SNP distribution along chromosomes, minor allele frequencies, and pairwise nucleotide diversity were estimated using the VCFtools program (Danecek et al., 2011). Linkage disequilibrium was determined for the entire WASAP and for each country's germplasm separately. Linkage disequilibrium was estimated based on the pair‐wise correlation coefficient (*r* ^2^) among SNPs with MAF > .05 (to reduce computational burden) in a window of 500 kb using the PopLDdecay package (Zhang, Dong, Xu, He, & Yang, 2019). The *smooth.spline* function in the R program (R Core Team, 2016) was used to fit LD decay measured as the distance by which *r* ^2^ decreased to half from its original value. The fixation index (F_ST_) genetic differentiation in the WASAP was determined according to botanical type and country of origin using the pair‐wise F_ST_ method based on Weir and Cockerham weighted F_ST_ estimate in the VCFtools.

### 2.4 Genetic structure analysis

The genome‐wide SNP variation of the collection was assessed based on the principal component analysis (PCA) using the *snpgdsPCA* function of the R package *SNPRelate* (Zheng et al., 2012). The accessions of WAS‐GRIN and GDP were included in the analysis to robustly determine principal axes of genome‐wide SNP variation and clustering of the WASAP along other germplasms. The combined dataset consisted of a subset of 103,871 (100K) high‐quality SNP markers with a high level of polymorphism (MAF > .1). The GDP accessions were used to determine the principal components (PCs) of genome‐wide variation. These PCs were then used to estimate the genome‐wide variation of the WASAP and WAS‐GRIN accessions.

The number of ancestral populations and ancestry fractions in the WASAP were determined using ADMIXTURE (Alexander, Novembre, & Lange, 2009). The imputed SNP dataset was LD pruned using PLINK 1.9 (Purcell et al., 2007) to reduce redundancy of SNPs that are in high LD because such SNPs provide the same genetic information. Ancestral populations and ancestry fractions were determined using the pruned SNP dataset (60,749 independent SNPs) for *K* = 2–20 populations using default settings of ADMIXTURE. A fivefold cross‐validation and 2,000 iterations were performed to identify the optimal *K*. We defined the optimal *K* as the minimum *K*‐value where cross‐validation error no longer decreased substantially. Accessions were assigned to subpopulations based on 0.7 membership threshold. The neighbor‐joining distance‐based clustering method was used to assess the genetic relationship among WASAP ancestral populations, WAS‐GRIN, and GDP. The genetic distance matrix was generated based on the 100K SNP set using TASSEL 5 (Bradbury et al., 2007) and the results were plotted using *ape* R package (Paradis, Claude, & Strimmer, 2004).

### 2.5 Field phenotyping

Field phenotyping of a subset of 572 WASAP accessions (based on seed availability) was conducted at the Bambey Research Station, Centre National de Recherches Agronomiques (14.42° N, 16.28° W) in Senegal during the growing season of 2014. Two experiments, early‐sown date at the beginning of the rainy season–normal planting date (Hiv1) and late‐sown postflowering drought‐stressed–planted 30 d later (Hiv2), were performed. A randomized incomplete block design (augmented block design) was performed with 30 blocks of 24 entries each following a column–row field layout for spatial data analysis. Each block contained 19 genotypes and five check varieties. The five checks were randomly assigned into each block. Each genotype was assigned only once in each experiment. Each genotype was planted in one row of 3 m surrounded by one row of fill material (IRAT 204 variety) on both sides. There was 60 cm of space between rows and 20 cm of space between plants or hills within a row. Ten days after planting, genotypes were thinned to maintain only one plant per hill. Days to flowering (DFLo) was measured as the day when 50% of plants within a plot flowered. Plant height (PH) was measured as the average distance from the soil to the tip of the panicle of three plants per plot.

### 2.6 Statistical analysis of phenotypic data

Phenotypic variation was analyzed using the R program. Spatial analysis was performed to verify the effect of spatial variation across each experiment based on the check varieties within blocks using the *SpATS* package (Rodríguez‐Álvarez, Boer, van Eeuwijk, & Eilers, 2018). The genotype‐adjusted mean value was determined after correcting for significant spatial variability. Variance components were estimated by fitting the mixed linear model with random effects for all genotypes (G), environment (E), and G × E interaction effects using the *lme4* package (Bates, Mächler, Bolker, & Walker, 2015). The significance levels of variance components were determined using the *RLRsim* package (Scheipl, Greven, & Küchenhoff, 2008). Broad‐sense heritability (*H* ^2^) was estimated for each trait from the estimated variance components following the formula proposed by (Piepho & Möhring, 2007) for unbalanced data:

$$\;H^{2}=\frac{\sigma_{G}^{2}}{\sigma_{G}^{2}\;+\;\bar{\nu }/2}\;$$

where $\sigma_{G}^{2}$ is the genotypic variance, $\bar{\nu }$ is the mean variance of a difference of two adjusted means. The early and late planting date experiments were considered as two environments. Phenotypic correlations among traits were calculated using the Pearson correlation of the *PerformanceAnalytics* package (Peterson et al., 2014). BLUP values for each trait were calculated by combining data from both environments. Tukey's Honestly Significant Difference (TukeyHSD) in the *Agricolae* package (Mendiburu, 2009) was used to test the difference of genotype performance between environments.

### 2.7 Genome‐wide association studies

Genome‐wide association studies were performed using the general‐linear model (GLM) in the GAPIT R package (Lipka et al., 2012) and multi‐locus mixed model (MLMM) in the *mlmm* package (Segura et al., 2012). The MLMM stepwise regression model was used to account for background effects. The GLM does not account for background effects (population structure and kinship) but shows most significant associations. In contrast, the mixed‐model method implemented in MLMM accounts for background effects but increases false‐negative associations. These different methods were used as complementary. We did not consider looking at nominal *p*‐value, as we are aware that the GLM results are generally inflated. Our goal was to test the hypothesis that some of the top associations would colocalize with known candidate genes. Bayesian information criterion and LD iteratively nested keyway (BLINK) (Huang, Liu, Zhou, Summers, & Zhang, 2019) was also used to verify overlapping associations with GLM and MLMM methods.

A total of 130,709 SNPs with MAF > .02 were used for the GWAS analysis. We use SNPs with MAF > .02 because moderately rare variants can contribute to phenotypic variation (Hernandez et al., 2019; Peloso et al., 2016). The first three PCs generated from TASSEL 5 and kinship matrix from GAPIT were used to account for polygenic background effects in the MLMM analysis. The GWAS were performed using BLUP values of DFLo and PH across two environments and for each environment. Linkage disequilibrium heatmaps of genomic regions surrounding GWAS QTL were constructed using the *LD heatmap 0.99‐4* package (Shin, Blay, McNeney, & Graham, 2006). All figures were produced with the R program. The effect size and proportion of phenotypic variance explained by associated QTL were determined using linear regression and ANOVA. Background effect was accounted for using ADMIXTURE ancestry fractions used as fixed‐effect covariates. Candidate gene colocalization with QTL was carried out using an a priori candidate genes and loci list, including known sorghum genes and orthologs of rice (*Oryza sativa* L.) and maize (*Zia mays* L.) for adaptive traits, previously described (Faye et al., 2019). Because candidate genes were a priori defined, we used a liberal cutoff of 500 kb to determine colocalization between association signals and candidate genes. The Sorghum QTL Atlas (Mace et al., 2019) was used to compare QTL identified in the current study to QTL from previous studies.

## 3 RESULTS

### 3.1 Genome‐wide SNP variation of the West African sorghum association panel

**FIGURE 1:**
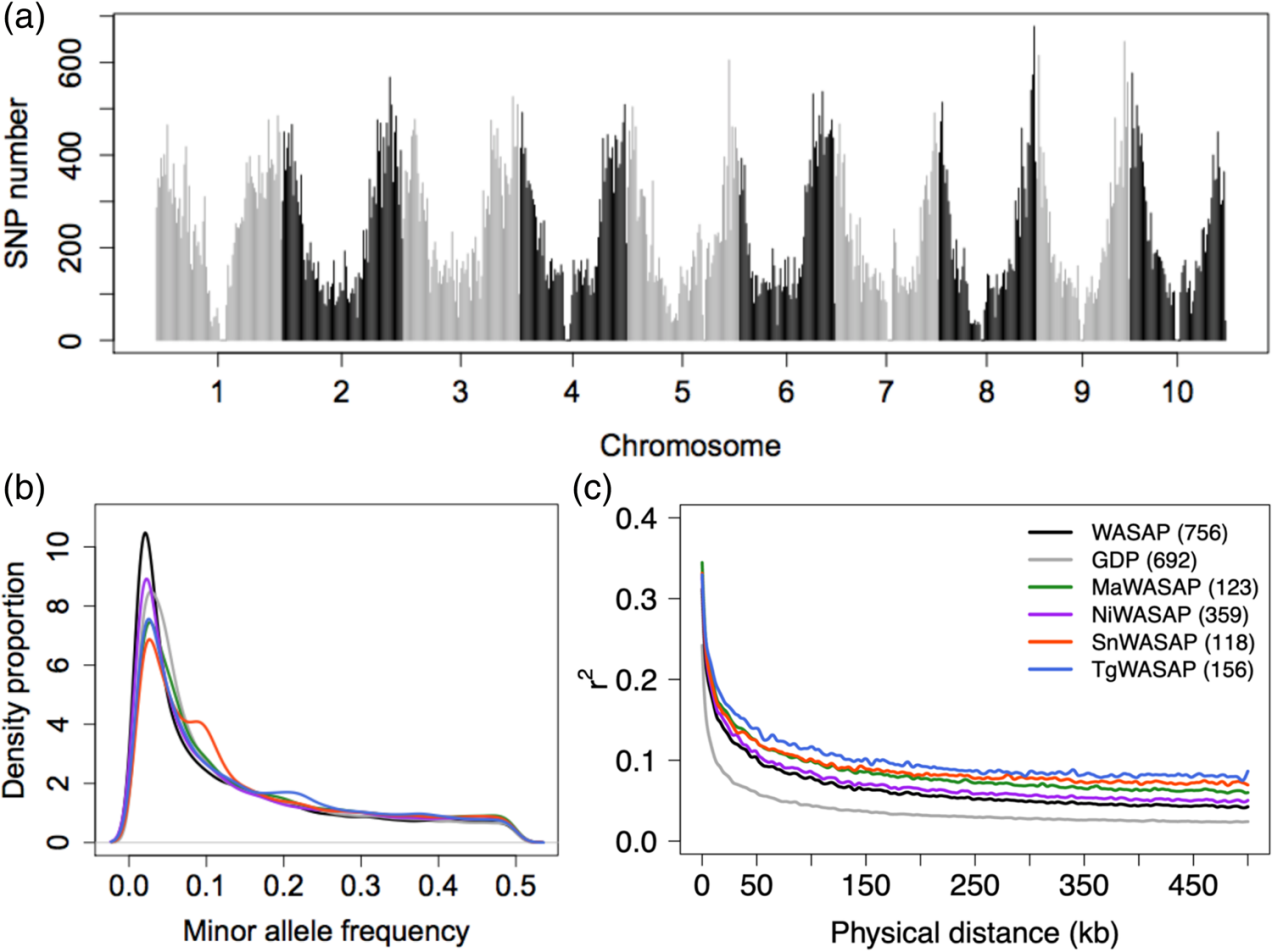
Genome‐wide single nucleotide polymorphism (SNP) variation in the West African sorghum association panel (WASAP) and global sorghum diversity panel (GDP). (a) Distribution of the SNP data across the 10 sorghum chromosomes in the WASAP. (b) Minor allele frequency distribution and (c) linkage disequilibrium decay along the genome of the SNP data in the whole WASAP, within countries of origin in WASAP–Mali (MaWASAP), Niger (NiWASAP), Senegal (SnWASAP), and Togo (TgWASAP) and in the global sorghum diversity panel (GDP)

The GBS library sequencing yielded a total of ∼258 million single‐end sequencing bar‐coded reads. After trimming all reads down to 64 bp, ∼4.5 million unique tags were obtained. A final data set of 159,101 high‐quality SNPs was maintained after removing SNPs with >20% missing data, MAF < .01, and keeping only biallelic SNPs. The SNPs were distributed across the genome with a higher number of SNPs in pericentromeric regions relative to centromeric regions (Figure 1a). We determined if the 159,101 GBS SNPs have potential impacts on protein‐coding sequences based on the sorghum reference sequence v.3.1. Approximately 13% of the SNPs were found in genic regions including 7,689 missense (72%), 411 (4%) nonsense, and 2,603 (24%) silent‐point mutations (Supplemental Figure S1). Approximately 43, 22, and 22% of the SNPs were located in intergenic, downstream, and upstream regions of genes, respectively.

Since four of five sorghum botanical types are represented in the WASAP, we hypothesized that it captures much of the genetic diversity found in the GDP. As predicted, the estimate of average pairwise nucleotide diversity (π) was only slightly less (18%) in the WASAP (.00045) than in the GDP (.00055). Little variation in π was observed among the four countries of origin (Niger, .00046; Mali, .00049; Senegal, .00050; and Togo, .00047). The WASAP had a higher proportion of rare alleles (e.g., 0.01–0.05) than the GDP (Figure 1b). Within the WASAP, the Senegal accessions had the lowest rare allele proportion and the highest intermediate allele proportion followed by Togo and Mali. Linkage disequilibrium decayed to background level (*r* ^2^ < .1) by ∼50 kb in the WASAP vs. ∼15 kb in the GDP (Figure 1c). As expected, LD decay within countries of origin in the WASAP was higher than that in the whole WASAP. Linkage disequilibrium decayed to background by ∼60, ∼90, ∼90, and ∼160 kb in Niger, Senegal, Mali, and Togo accessions, respectively.

### 3.2 Genetic differentiation by botanical types and geographic origin

Based on the hypothesis that sorghum genetic diversity is structured primarily by botanical type, we predicted high F_ST_ genetic differentiation would be observed among botanical types than among countries of origin in the WASAP. High F_ST_ genetic differentiation of .16 was observed among the six classes, comprising the majority of the panel including guinea, caudatum, durra, bicolor, guinea margaritiferum types, and the intermediate form durra–caudatum (Supplemental Table S1). Surprisingly, a high F_ST_ value was observed between durra–caudatum and caudatum (F_ST_ = .22) or durra–caudatum and durra (F_ST_ = .20). The F_ST_ value among the four countries of origin was moderate (F_ST_ = .09) (Supplemental Table S1).

**FIGURE 2:**
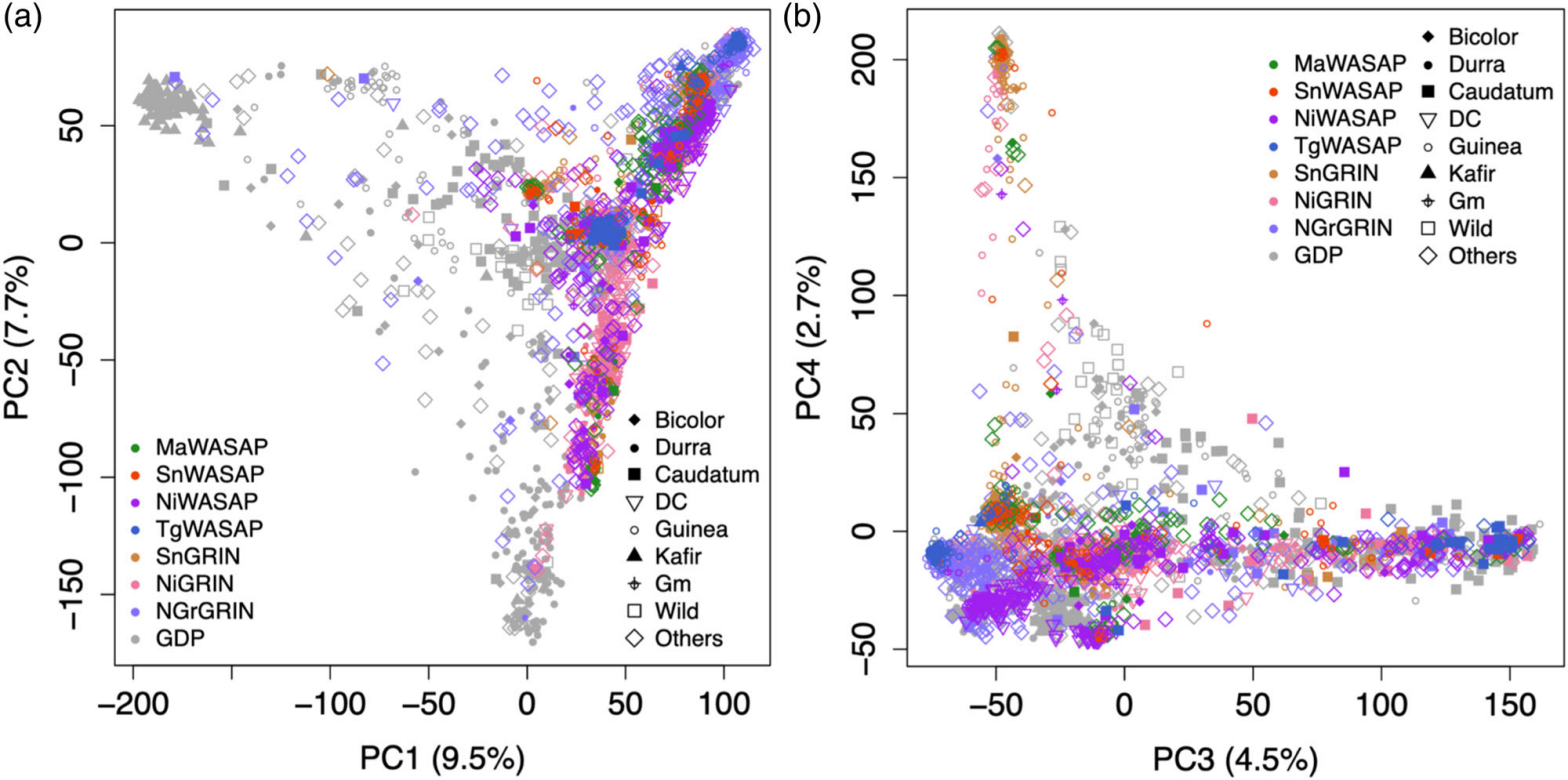
Principal component analysis of genome‐wide single nucleotide polymorphism (SNP) variation. Scatterplots of the (a) first and second axes and the (b) third and fourth axes of genome‐wide SNP variation in the West African sorghum association panel (WASAP) in relationship with other West African sorghums in GRIN and global sorghum diversity panel. The color codes indicate country of origin for WASAP accessions (MaWASAP, Mali; NiWASAP, Niger; SnWASAP, Senegal, and TgWASAP, Togo), the West African accessions in GRIN (SnGRIN, Senegal, Gambia and Mauritania; NiGRIN, Niger; NGrGRIN, Nigeria), and the global sorghum diversity panel (GDP). The symbols indicate botanical types where DC and Gm correspond to durra–caudatum intermediate and guinea margaritiferum types, respectively

Next we used PCA to characterize the genomic variation of the WASAP with respect to botanical type and origin vs. with WAS‐GRIN and GDP accessions. The first two PCs explained a high proportion of genome‐wide SNP variation (17%) and differentiated the caudatum, durra, guinea, and kafir accessions (Figure 2a). We predicted that the majority of the WASAP accessions will be clustered with guinea, caudatum, and durra accessions from the same geographic origin in the GDP. The majority of the WASAP accessions overlapped with their corresponding types, guinea, durra, and caudatum clusters of GDP along the PC2. A substantial variation was explained by both PC3 vs. PC4 (7.3%) (Figure 2b). The durra–caudatum intermediate types in the WASAP and WAS‐GRIN clustered between durra, caudatum, and guinea clusters in the GDP.

### 3.3 Ancestral fractions and population structure

**FIGURE 3:**
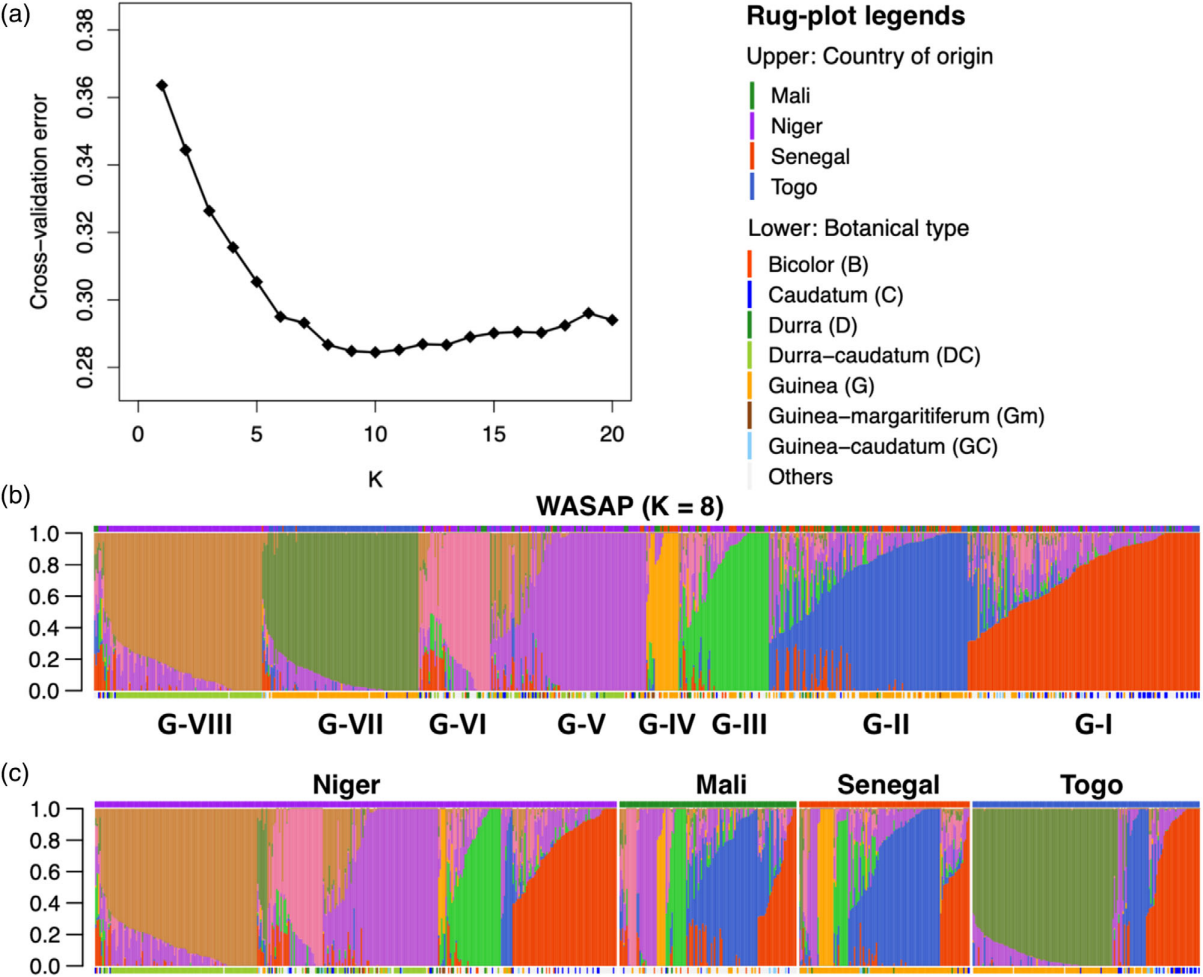
Genetic ancestry analysis of the West African sorghum association panel (WASAP). (a) Fivefold cross‐validation error from the ADMIXTURE model using 60,749 single nucleotide polymorphisms for *K* = 2–20. Ancestral genetic groups of the WASAP at *K* = 8 ancestral populations (b) ordered by ancestry fraction and (C) ordered by country then by ancestry fraction. Vertical bars represent ancestry fraction from the eight ancestral populations (G‐I to G‐VIII) indicated with a different arbitrary color for each population (Note, ancestral population color coding in the vertical bars does not correspond to the rug‐plot color coding). Upper rug plots indicate countries of origin. Lower rug plots indicate botanical type (‘Others’ include rare intermediate types and accessions of unknown botanical type). Ancestry fractions for each accession are in Supplemental Data S1

To determine the ancestral populations and ancestry fractions for each accession, we used the Bayesian model‐based method ADMIXTURE. Based on fivefold cross‐validation error, the optimum number of ancestral populations was eight (Figure 3a). The accessions classified morphologically as guinea (orange lower rug plot) corresponded to three genetic groups (G‐II, G‐IV, and G‐VII) (Figure 3b; Supplemental Data S1). The accessions classified morphologically as durra–caudatum intermediates (mostly from Niger; green lower rug plot) corresponded to two genetic groups (G‐V and G‐VIII). Accessions classified morphologically as caudatum (blue lower rug plot) corresponded to genetic group G‐I. Using 0.7 ancestry fraction as a threshold, 71% of accessions could be assigned to a subpopulation, while 29% would be considered admixed. The greatest putative contribution to genetic admixture was from G‐V (purple bars), with ancestry fraction present in all other subpopulations. The F_ST_ value among ancestral populations averaged .39, with a range of .25–.61 (Supplemental Table S2).

**FIGURE 4:**
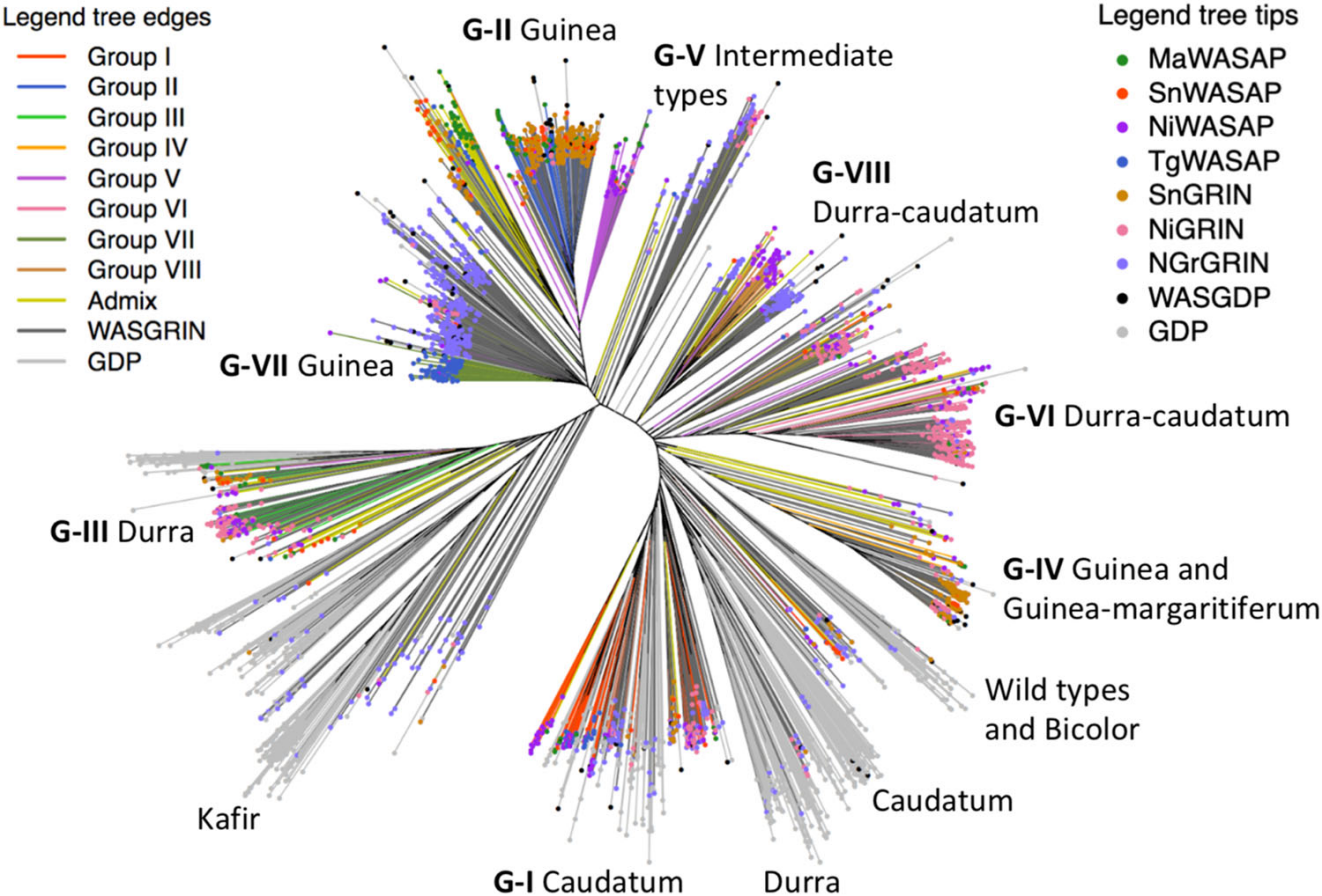
Neighbor‐joining analysis of the West African sorghum association panel (WASAP). Clustering of the WASAP accessions (MaWASAP, Mali; NiWASAP, Niger; SnWASAP, Senegal, and TgWASAP, Togo) in relationship with other West African sorghums in GRIN (SnGRIN, Senegal, Gambia, and Mauritania; NiGRIN, Niger; NGrGRIN, Nigeria) and global sorghum diversity panel (GDP). The color coding of the tree edges is based on the ADMIXTURE ancestral populations (G‐I to G‐VIII, including admixed accessions) of the WASAP. The edges in yellow, dark gray, and light gray represent admixed WASAP accessions (<0.6 ancestry fraction), West African sorghum in USDA–GRIN (WASGRIN) accessions, and GDP accessions, respectively. The color coding of the tree tips indicate accessions origin, with black tips indicating West African sorghum accessions in the GDP (WASGDP)

Next, we considered the extent to which germplasm of each country is distinct from the germplasm of other countries (Figure 3c). Each country's germplasm included multiple genetic groups. Most of the ancestral populations were found in each country, except in Togo, where only three genetic subpopulations (G‐I, G‐II, and G‐VII) were clearly defined. The G‐IV genetic group was specific to Senegal and Mali, while G‐VI was specific to Niger and Mali. Genetic groups G‐VII and G‐VIII were specific to Togo and Niger, respectively. Neighbor‐joining analysis recapitulated the country‐level ancestry structure (see color‐coded tips) (Figure 4; Supplemental Figure S2). Genetic similarities were observed between WASAP ADMIXTURE genetic groups and other West African sorghums (WAS‐GRIN and WAS‐GDP) from the same geographic origin and GDP accessions according to botanical type and geographic origin. The West African sorghums clustered with their corresponding types in the GDP. The guinea and durra–caudatum accessions of WA clustered generally distinctly from the majority of GDP accessions.

### 3.4 Phenotypic variation in the WASAP

A total of 572 accessions of the WASAP and five check lines were evaluated for agronomic traits under rainfed conditions. We hypothesized that the phenotypic variation in the WASAP is due to genetic effects, appropriate for mapping with GWAS. Large phenotypic variation was observed for both DFLo (21%) and PH (35%). Significant genotypic (G) variation and G × E interaction effects were observed (Table 2). Broad‐sense heritability was high across the two environments, with values ranging from .71 for PH to .88 for DFLo (Table 2). For each trait, phenotypic variation was similar between the two environments for each main botanical type (guinea, caudatum, and durra) and country of origin (Supplemental Table S3). In both environments, guinea types had higher average DFLo than caudatum and durra types. Accessions from Togo had higher average DFLo than accessions from other countries. Days to flowering were significantly higher in Hiv1 (Tukey's honest significant difference, *p* < .0001; Supplemental Figure S3), but significantly correlated based on the 1:1 ratio, between Hiv1 and Hiv2 (*r* ^2^ = .75, *p* < .0001) (Supplemental Figure S4A). The relationship holds within country of origin, for instance for accessions from Togo (*r* ^2^ = .77, *p* < .0001) and Senegal accessions (*r* ^2^ = .78, *p* < .0001) (Supplemental Figure S4B). Days to flowering and PH were significantly correlated in each environment (Supplemental Figure S5).

**TABLE 2:** Descriptive statistics and phenotypic variation across early (Hiv1) and late (Hiv2) planting date experiments under rainfed conditions

				Variances			
Trait	Range	Mean ± SD	CV	Genotype (G)	Environment (E)	G×E	Broad‐sense heritability
			%				
Days to flowering	40–128	78 ± 15	21	79***	1ns^†^	9***	.88
Plant height (cm)	65–410	214 ± 75	35	57***	3*	20***	.71

^*^Significant at the .05 probability level. ^***^Significant at the .001 probability level. †ns, nonsignificant.

### 3.5 Genome‐wide association studies for flowering time and plant height

**FIGURE 5:**
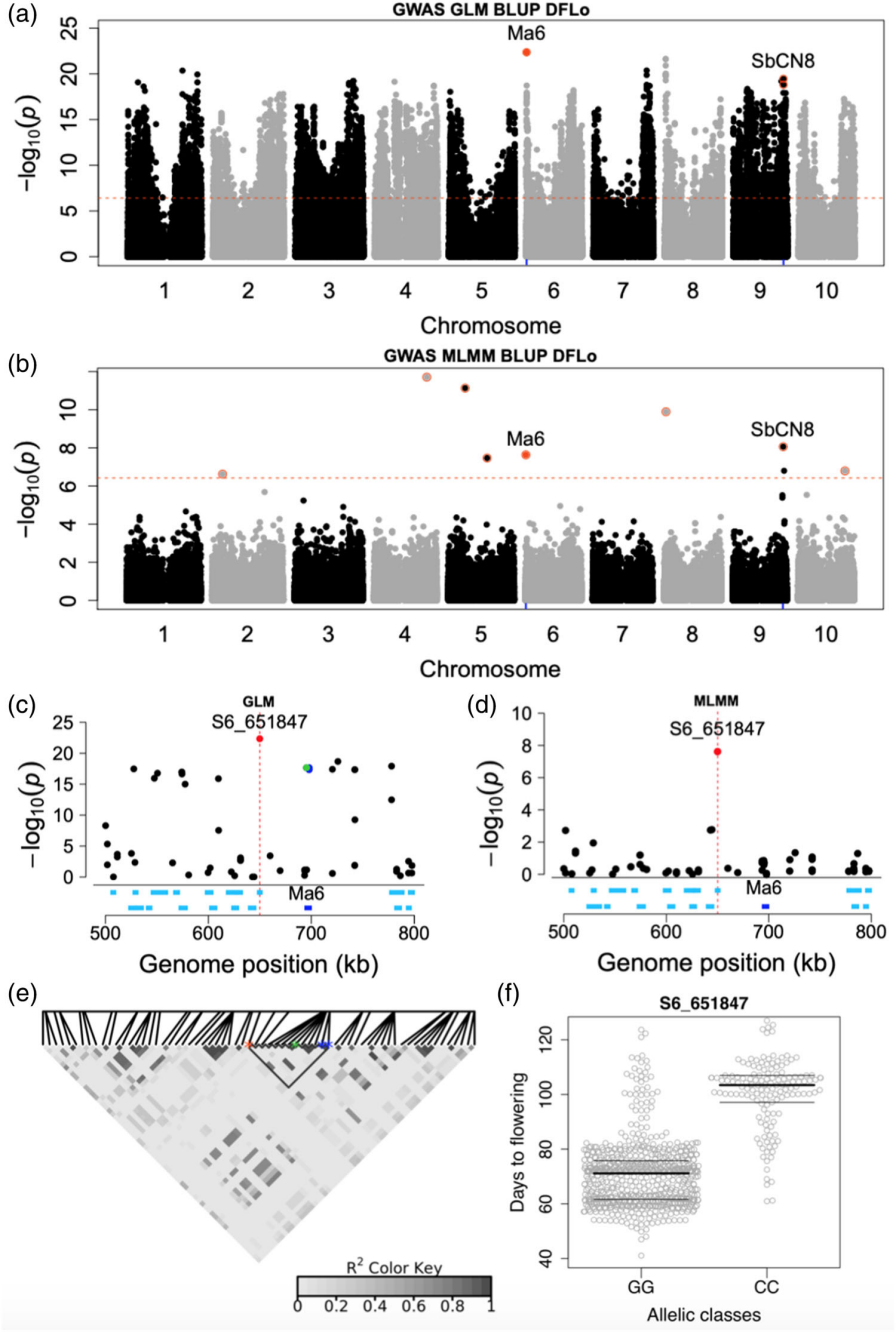
Genome‐wide association studies for days to flowering (DFLo) under rainfed conditions. Manhattan plots of DFLo based on (a) the general linear model (GLM) and (b) the multi‐locus mixed‐linear model (MLMM). The horizontal red dashed line represents the Bonferroni significance threshold at .05. The rug plots indicate the position of colocalizing candidate genes, *Ma6* and *SbCN8* with quantitative trait loci (QTL). Regional Manhattan plot of a 150‐kb region on chromosome 6 around the QTL S6_651847 that colocalizes with *Ma6* from (c) GLM and (d) MLMM. The green and blue peaks are single nucleotide polymorphism QTL at 160 bp from and within *Ma6*, respectively. The dark blue segment indicates the genomic position of *Ma6*. (e) Linkage disequilibrium heatmap of a 150‐kb region surrounding the QTL S6_651847. The red, green, and blue asterisks indicate the S6_651847, SNPs at 160 bp from *Ma6* and within *Ma6*, respectively. (f) Days to flowering across planting dates by allelic classes of the QTL S6_651847

We assessed the effectiveness of the genome‐wide SNP data for genetic dissection of complex quantitative traits using GWAS. For DFLo BLUPs, the GLM model identified many significant associations at Bonferroni correction .05 (Figure 5a). A leading QTL was identified near *Ma6*/*Ghd7* candidate gene between SNPs S6_651847 (top association in the region, ∼45 kb away) and S6_699843 (within gene). A second leading QTL was identified between S9_54917833 and S9_54968379 at 43 kb and 4 kb from *SbCN8* candidate gene, respectively (Supplemental Table S4). The GLM showed inflation of *p*‐values with many significantly associated SNPs. The MLMM model identified eight QTL at Bonferroni threshold (3.8 × 10^−7^). Some of these QTL colocalized with known candidate genes *Ma6* at 45 kb (S6_651847) and *SbCN8* at 381 kb (S9_55345348) (Table 3). The QTL near *Ma6*, S6_651847, was the top peak in the region in both GLM and MLMM and was at one gene away from *Ma6* (Figure 5c and 5d). Linkage disequilibrium between the QTL, S6_651847, and SNPs near or within *Ma6* locus was moderate (Figure 5e). After controlling for the population structure, the association of S6_651847 had an estimated effect size of 29 d and proportion of variance explained (PVE) of 25% (Table 3; Figure 5f). The BLINK model identified the same QTL, presented above, near *Ma6* and *SbCN8* (Supplemental Figure S6A). For DFLo in each environment, the GLM identified the same QTL as the top associations in the regions of *Ma6* and *SbCN8* in both Hiv1 and Hiv2, but the BLINK model identified only the QTL at *SbCN8* in Hiv1 (Supplemental Figures S6B and S6C).

**TABLE 3:** Quantitative trait loci (QTL) associated with days to flowering best linear unbiased predictions across early and late planting date experiments using the multi‐locus mixed‐linear model (MLMM)

QTL SNP^a^	MLMM *p*‐value	MAF^b^	Effect size	PVE^c^	MLM^d^ *p*‐value	Distance to locus	Locus name
				%		kb	
S4_57407080	<10^‐12^	.02	10	7	.002	–	–
S5_18513685	<10^−12^	.10	12	9	.0008	–	–
S8_2206437	<10^−10^	.04	28	11	.0001	–	–
S9_55345348	<10^−9^	.04	15	9	.0005	381	*SbCN8*
S6_651847	<10^−8^	.25	29	25	<10^−11^	45	*Ma6*
S5_42404204	<10^−8^	.12	6	16	.0001	–	–
S10_51083132	<10^−7^	.28	7	5	.03	–	–
S2_11509143	<10^−7^	.04	5	0.3	.6	–	–

^a^ Digit before and after underscore indicates chromosome number and single nucleotide polymorphism (SNP) position on the genome, respectively.

^b^ MAF, minor allele frequency.

^c^ ADMIXTURE ancestry fractions at *K* = 8 were used as fixed effect covariates; PVE, proportion of variance explained.

^d^ MLM, mixed‐linear model.

**FIGURE 6:**
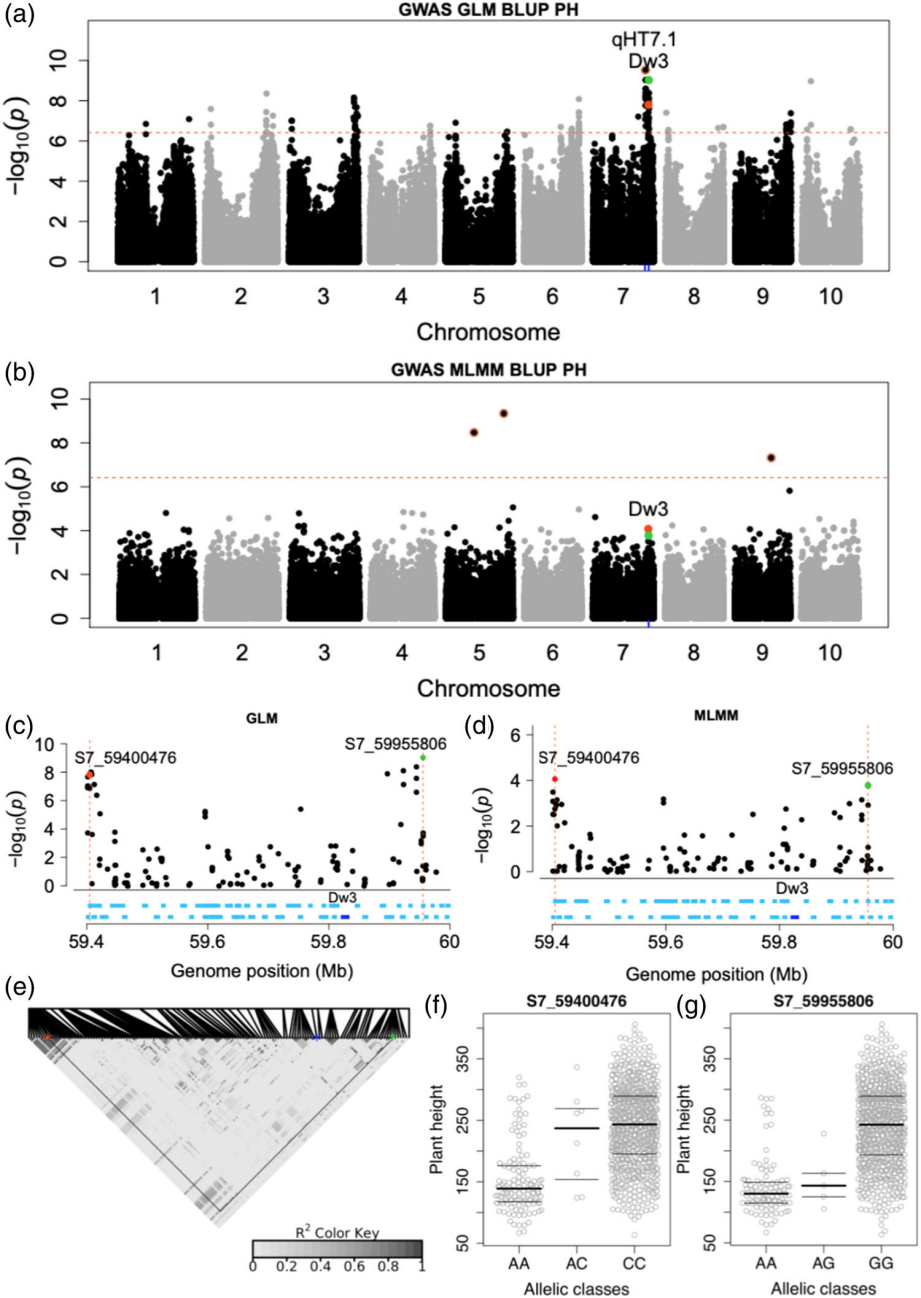
Genome‐wide association studies for plant height (PH) under rainfed conditions. Manhattan plots of PH based on (a) the general linear model (GLM) and (b) the multi‐locus mixed‐linear model (MLMM). The horizontal dashed line represents the Bonferroni significance threshold at .05. Rug plots on chromosome 7 indicate the position of the candidate gene, *Dw3* and *qPH7.1*. Regional Manhattan plot of a 600‐kb region on chromosome 7 surrounding the quantitative trait loci between S7_59400476 and S7_59955806 that colocalizes with *Dw3* from (c) GLM and (d) MLMM. The red and green peaks are top single nucleotide polymorphisms (SNPs) in MLMM and GLM, respectively. The dark blue segment indicates the genomic position of *Dw3*. (e) Linkage disequilibrium heatmap of genomic region between SNPs S7_59400476 and S7_59955806. The red, green, and blue asterisks indicate the S7_59400476, S7_59955806, and a SNP within *Dw3*, respectively. Days to flowering across planting dates by allelic classes of SNPs (f) S7_59400476 and (g) S7_59955806

For PH BLUPs, the GLM model identified several associations (Figure 6a). The top association, S7_56232413, overlapped with the height QTL *qHT7.1* (Bouchet et al., 2017; Li, Li, Fridman, Tesso, & Yu, 2015). A second QTL was identified between SNPs S7_59955806 (top association in the region) and S7_59402662 located at 125 and 419 kb from the *Dw3* a priori candidate gene, respectively (Supplemental Table S5). The MLMM identified three QTL at the Bonferroni threshold (Figure 6b; Supplemental Table S6). A putative SNP QTL, S7_59400476, was identified 421 kb away from *Dw3*, though below the Bonferroni threshold. After accounting for confounding population structure effect, the MLMM QTL still had significant estimated effect sizes and contributed to high PVE for PH (Supplemental Table S6). Linkage disequilibrium between S7_59955806 and S7_59400476 was high (though these two SNPs are separated by 555 kb from each other) but weak between these SNP QTLs and a variant in *Dw3* locus (Figure 6e). Alleles at both SNP QTL were associated with height differences of accessions across planting dates (Figure 6f and 6g). The association of S7_59400476 with PH, which had the highest allelic effect estimate (73 cm) and PVE (41%), was confirmed using MLM (*p* < 10^−13^) (Supplemental Table S6). The BLINK model identified the same QTL near *Dw3* and *qHT7.1* (Supplemental Figure S7A). For PH in each environment, the GLM identified the two QTL in the regions of *Dw3* and *qHT7.1* in both Hiv1 and Hiv2, but the BLINK model identified these two QTL only in Hiv2 (Supplemental Figure S7B and S7C).

## 4 DISCUSSION

### 4.1 Developing a high‐quality regional genomic resource

In the present study, we assembled 756 sorghum accessions from West African sorghum germplasm and characterized the genome‐wide SNP variation of the panel. We demonstrated that this genome‐wide SNP dataset is of sufficient quality for genomic and quantitative genetic analyses suitable for crop improvement through genomics‐assisted breeding. The strict data filtering criteria used before and after genotype imputation provided a final dataset with reduced number of SNPs (*n* = 159,101) many of which have impacts on protein‐coding sequences (Supplemental Figure S1A).

The quality control of the SNP dataset matched our expectations, as the F_ST_, PCA, and neighbor‐joining analyses (Figures 2 and 4; Supplemental Figure S2) confirmed the expected structure of sorghum by botanical type and geographic region (Bouchet et al., 2017; Lasky et al., 2015; Morris et al., 2013; Wang, Hu, Upadhyaya, & Morris, 2019). The validity of the SNP dataset was further confirmed based on GWAS with the identification of QTL (Figures 5 and 6) that colocalized with known candidate loci: *Ma6* and *SbCN8* (Murphy et al., 2014; Yang et al., 2014) for days to flowering and *qHT7.1* and *Dw3* (Li et al., 2011, 2015; Multani et al., 2003) for plant height. Although regional diversity panels could limit confounding effects as a result of population structure in GWAS, in this case, the strong population structure in the WASAP appeared to increase false‐positive associations in the GLM (Brachi, Morris, & Borevitz, 2011). However, using the stepwise regression in MLMM though helped to control the inflation of *p*‐values observed in the GLM (Figure 5 and 6).

Resolution of GWAS depends on LD decay across the genome (Korte & Farlow, 2013; Slatkin, 2008). The LD decay range was generally short in the accessions of the GDP analyzed in the present study (Figure 1c) compared with the long LD range in GDPs reported in previous studies. In sorghum, studies have reported variation in LD decays from short (10–15 kb) to moderate (50–100 kb) (Bouchet et al., 2012; Hamblin et al., 2005) to ∼150 kb in some studies that used higher marker density with larger genome coverage and larger population size (Mace et al., 2013b; Morris et al., 2013). Different marker systems, genome coverage, and population size differences influence LD estimates, making LD decays comparison difficult among studies. The shorter LD range observed in the GDP in this study could be explained by the exclusion of North American breeding lines that share many common haplotypes (Klein et al., 2008).

As expected, LD decay range was longer in the WASAP than the GDP. This result is mainly due to the lesser genetic diversity at the regional scale than the global sorghum diversity. Within the WASAP, patterns of LD decay were similar among countries of origin but the rates of LD decay varied among populations. The shorter LD in Niger accessions, which is comparable with LD decay of the whole WASAP, reflects the strong genetic structure in Niger where all four basic botanical types and eight ancestral populations are present (Figure 3c). In contrast, the longer LD range in Togo accessions is consistent with the limited number of genetic subpopulations observed in Togo, with only two guinea and one caudatum subpopulations.

### 4.2 Insights into hierarchical population structure in West African sorghum

Sorghum genetic studies have identified population structure by botanical type and geographic region at regional scale (Bouchet et al., 2012; Deu et al., 2006; Morris et al., 2013; Wang et al., 2019) and at country level in the Senegal and Niger germplasm (Deu et al., 2008; Faye et al., 2019). The genetic diversity of the WASAP was structured by botanical type within each country of origin (Figures 3 and 4). This finding is congruent with the F_ST_ analysis (Supplemental Table S1) where botanical type and country of origin contributed to high and moderate genetic differentiation, respectively.

The guinea type in the WASAP was split into three major subgroups (Figure 4). One subgroup was formed by Senegal and Mali accessions, a second subgroup formed by Togo accessions (which clustered with Nigeria accessions), and a third subgroup that was more related to durra and durra–caudatum types, formed predominantly by Senegal accessions. This third group (G‐IV) clustered with guinea margaritiferum accessions from Niger and was more related to bicolor and wild sorghums in the GDP. Previous studies have found four main guinea subgroups; however, these subgroups were distinctly comprised of guineas from southern Africa, Asia, western Africa, and guinea margaritiferum (Deu et al., 2006). There were no significant genetic differences between WASAP and WAS‐GRIN subpopulations from the same geographic origin, suggesting that the globally accessible GRIN collections are representative of current germplasm in WA. In contrast, overall genetic differences were observed between West African accessions and GDP where few subpopulations were formed almost entirely by West African sorghum accessions (e.g., G‐V, G‐VI, and G‐VII; Figure 4), highlighting the distinctiveness of West African germplasm.

The high genetic diversity observed within each of the four countries of the WASAP (Figure 3c) is relevant for breeding programs in the region. Within the WASAP, all eight ancestral populations were found in the germplasm of each country, except in the Togo germplasm, which appeared to be less diverse based on results of LD decay, population structure, and neighbor‐joining tree analyses. Altogether, the genetic diversity of WA sorghum germplasm is hierarchically structured by botanical type and subpopulation within botanical type with many subpopulations distributed across countries.

### 4.3 Suitability of genomic resources for GWAS

To establish the utility of our genome‐wide SNP dataset for GWAS, we carried out GWAS for DFLo and PH and demonstrated colocalization of QTL with known genes from previous studies. While flowering time genes and natural variants have been characterized in the U.S. sorghum (Casto et al., 2019; Murphy et al., 2014), the genetic basis of the substantial photoperiodic flowering time variation in West African sorghum is not yet known (Bhosale et al., 2012). The QTL S6_651847 near *Ma6* (*Gdh7*) (Murphy et al., 2014) highly contributed to the proportion of phenotypic variation of flowering time. This QTL was mapped by GLM, MLMM, and BLINK methods across environments (Figure 5c and 5d; Supplemental Figure S6A), indicating that *Ma6* might be a major flowering time gene in the WA sorghum germplasm. The large effect of the QTL near *Ma6* is consistent with the hypothesis that *Ma6* contributes to photoperiodic flowering time variation in sorghum (Murphy et al., 2014) including WA sorghum. Only two SNPs, which were in low LD with the QTL S6_651847 (Figure 5e), were found within the *Ma6* locus. Several of the other identified QTL overlapped with flowering time QTL found in other studies based on the sorghum QTL atlas (Mace et al., 2019) (Supplemental Table S7).

Our findings are consistent with the hypothesis that a substantial oligogenic component underlies flowering time variation in West African sorghums, suggesting that photoperiodic flowering could be selected using markers from large‐effect QTL. The two MLMM height QTL (S5_30001948, and S9_38942669) accounted for 13.3 and 20.9%, respectively, of height variation (Supplemental Table S6) but were not colocalized (within 500 kb) with known height genes. The major‐effect QTL from this study were mapped using a diversity panel but their effects on breeding populations and different elite backgrounds across West African environments are unknown. These major‐effect QTL would need to be validated with multi‐year phenotypic data and other approaches, such as multi‐parent linkage mapping and near‐isogenic lines. Following validation, these height and flowering QTL could be useful for marker‐assisted selection to recover locally adaptive plant types across botanical types within WA or during prebreeding with exotic trait donors (Yohannes et al., 2015). Given the positive findings for height and flowering time, we anticipate this resource will be suitable for characterization and mapping of other complex traits of interest to West African breeding programs.

### 4.4 Implications for sorghum improvement in western Africa and globally

This study demonstrates that breeding populations in each of the four countries in WA harbor sufficient genetic diversity. The hierarchical population structure observed in the WASAP at country level suggests the existence of multiple ancestral populations within the country but similar across WA (Deu et al., 2008; Folkertsma et al., 2005; Sagnard et al., 2011). The increased kinship within each subpopulation enables the implementation of genomic selection within an individual subpopulation or a subpopulation across breeding programs. Otherwise, the strong population structure could lead to biased prediction accuracy as a result from allele frequency differences among subpopulations (Isidro et al., 2015). Although there are flowering time differences in the WASAP, Togo material can adapt to the growing season of other countries because, as a result of their photoperiod sensitivity, their maturity cycle would fit the growing season in those regions. For instance, Togo materials grown in common garden experiments in Senegal were able to mature on time in both sowing dates. This panel is suitable for assessing agronomic traits such as grain yield from caudatum cultivars, drought tolerance from durra and durra–caudatum cultivars, or grain quality and disease resistance from guinea cultivars.

This SNP dataset could be used by the genetics community in genomic prediction, genetic diversity and haplotype analyses, and GWAS to serve global sorghum breeding especially in combination with other regional genomic resources. Historically, West African sorghum germplasm has been useful for global sorghum breeding including durra and caudatum accessions that were sources of yellow endosperm and drought tolerance for U.S. breeding programs (Rosenow & Dahlberg, 2000). Whole‐genome resequencing would complement the GBS SNP dataset to more easily identify putative causal variants for adaptive traits in the West African germplasm (Bellis et al., 2020). The development of trait‐predictive markers could facilitate more rapid breeding of locally and regionally adapted sorghum varieties for the diverse stakeholders of WA.

## CONFLICT OF INTEREST

The authors declare no conflict of interest.

## AUTHOR CONTRIBUTIONS

Conceived and managed the study: GPM, DF, NC, AM, JFR. Contributed the genetic materials: NT, AM, EAA, CD, NC. Collected the data: SM, SB, FM, JMF, BS, EAA. Analyzed and visualized the data: JMF. Wrote the manuscript: JMF, GPM. All authors edited and approved the manuscript.

## Supporting information



Supplemental Figure S1. Genome‐wide single nucleotide polymorphisms in the WASAP.
Supplemental Figure S2. Neighbor‐joining tree of WASAP ancestral populations in relationship with other West African sorghums in GRIN (WAS‐GRIN) and the global sorghum diversity panel (GDP).
Supplemental Figure S3. Average performance values of check varieties within each planting date experiment (Hiv1 and Hiv2) under rainfed conditions.
Supplemental Figure S4. Flowering time differences of accessions between early (Hiv1) and late (Hiv2) planting date experiments under rainfed conditions.
Supplemental Figure S5. Phenotypic correlations for days to flowering and plant height in early (Hiv1) and late planting date experiments (Hiv2).
Supplemental Figure S6. GWAS for days to flowering (DFLo) in separate and across environments based on GLM and BLINK models.
Supplemental Figure S7. GWAS for plant height (PH) in separate and across environments based on GLM and BLINK models.
Supplemental Table S1. Pairwise weighted F_ST_ among botanical types and countries of origin. The four basic botanical types and the durra–caudatum types that composed the majority of WASAP were used for the F_ST_ analysis.
Supplemental Table S2. Pairwise weighted F_ST_ among ADMIXTURE ancestral populations.
Supplemental Table S3: Phenotypic variation in early (Hiv1) and late (Hiv2) planting date experiments among main botanical types and countries of origin.
Supplemental Table S4. Quantitative trait loci near *Ma6* and *SbCN8* candidate genes associated with DFLo BLUPs using the GLM.
Supplemental Table S5. Quantitative trait loci near *qHT7.1* and *Dw3* candidate genes associated with plant height BLUPs using the GLM.
Supplemental Table S6. Quantitative‐trait loci associated with plant height BLUPs across two combined environments using the MLMM.
Supplemental Table S7. List of GWAS QTLs, excluding those at *Ma6*, *SbCN8*, and *Dw3*, overlapping with published QTLs from other studies based on the sorghum QTL Atlas.
Supplemental Data S1. Country of origin and botanical type of accessions of the West African sorghum association panel, including ADMIXTURE ancestry fractions in populations at K = 8.

## Data Availability

The SNP and phenotypic datasets generated from this study are available in the Dryad Data Repository under accession https://doi.org/10.5061/dryad.k0p2ngf67.
